# Manipulation of Pluripotent Stem Cell Metabolism for Clinical Application

**DOI:** 10.1007/s40778-017-0073-9

**Published:** 2017-02-13

**Authors:** Shugo Tohyama, Sho Tanosaki, Shota Someya, Jun Fujita, Keiichi Fukuda

**Affiliations:** 1 Department of Cardiology, Keio University School of Medicine, 35 Shinanomachi Shinjuku-ku, Tokyo, 160-8582 Japan; 2 Department of Organ Fabrication, Keio University School of Medicine, 35 Shinanomachi Shinjuku-ku, Tokyo, 160-8582 Japan

**Keywords:** Embryonic stem cells, Induced pluripotent stem cells, Differentiation, Purification, Metabolism, Regenerative therapy

## Abstract

**Purpose of review:**

Pluripotent stem cells (PSCs) have the capacity to differentiate into various types of cells, and are promising cell sources for regenerative therapy and drug screening. However, to realize the clinical application of PSCs, a large number of highly qualified target cells must be stably prepared with low cost. To achieve this, great improvements in the reprogramming, differentiation, and elimination of residual PSCs will be necessary. In this review, we summarize the updated knowledge about metabolism in PSCs and its application.

**Recent findings:**

Recent studies have shown that PSCs have distinct metabolic profiles compared to differentiated cells. The metabolic profiles of PSCs are indispensable for the maintenance of pluripotency, self-renewal, differentiation capacity, and cell survival.

**Summary:**

Metabolic approaches show improved simplicity, scalability, and lower cost than conventional methods for differentiation and elimination of residual PSCs. Thus, manipulation of PSC metabolism will lead to new technologies to improve their efficiencies.

## Introduction

Pluripotent stem cells (PSCs), including embryonic stem cells (ESCs) [1] and induced pluripotent stem cells (iPSCs), can theoretically self-renew infinitely [2, 3] and differentiate into various types of cells, including cardiomyocytes, neurons, and hepatocytes. Based on their unique characteristics, the clinical application of PSCs such as in regenerative therapy and drug screening for numerous diseases has been proposed. However, for realization of their clinical application, a large number of cells should be stably prepared without requiring an enormous cost. There are many processes available to obtain the target cells, including reprogramming, differentiation, and elimination of residual PSCs from PSC-derived cells. Each process has been dramatically improved in recent years with the development of many technologies. With respect to the reprogramming process, use of a temperature-sensitive mutated Sendai virus with Yamanaka factors (OCT4, SOX2, KLF4, and c-MYC) was shown to substantially improve the efficiency of iPSC generation [4]. In addition, co-expression of maternal-specific factors in oocytes such as GLIS1 [5], TH2A/TH2B [6], and H1foo [7] with Yamanaka factors also enhanced the reprogramming efficiency in iPSC generation. With respect to pluripotency, human PSCs (hPSCs) have been shown to be capable of switching to the naïve state by use of a combination of small molecules, similar to mouse PSCs (mPSCs) [8–11]. Moreover, the efficiencies of differentiation into cardiomyocytes could be greatly improved with the use of recently identified small molecules rather than recombinant proteins [12, 13]. Furthermore, fluorescence-activated cell sorting (FACS)-based methods or toxin-based methods have been developed for the elimination of residual PSCs after differentiation [14–17]. However, more improvements are required to realize the clinical application of PSCs in terms of reducing the cost and enhancing the scalability.

PSCs show unique metabolic features to maintain their pluripotency and proliferative ability, and their metabolism changes dramatically during the processes of reprogramming and differentiation. Accordingly, methods to improve metabolic regulation should be of great benefit for enhancing the efficiencies of PSCs. Many powerful analysis tools such as metabolomics, proteomics, and transcriptomics have been developed to obtain metabolic profiles [18–20], which can be used to dramatically advance analyses of the metabolic features of PSCs and differentiated cardiomyocytes. Here, we introduce the updated knowledge about metabolism in PSCs and manipulation of their metabolism for clinical application.

### Metabolism for Self-Renewal and Pluripotency in PSCs

#### Metabolism in mPSCs

**Fig. 1:**
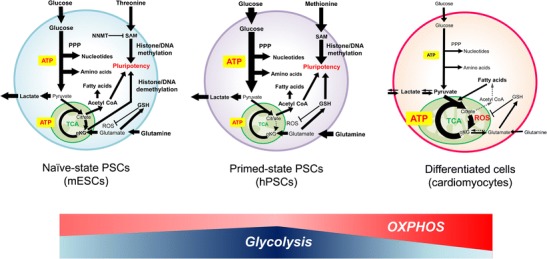
Metabolic features in PSCs and differentiated cardiomyocytes. Naïve-state PSCs like mESCs depend on glucose and glutamine metabolism. Glucose or glutamine-derived αKG is important for histone and DNA demethylation related with pluripotency. Primed-state PSCs like hPSCs highly depend on glycolysis compared to naïve-state PSCs due to higher HIF1α expression. In addition, hPSCs also depend on glutamine oxidation not only for ATP production but also for glutathione (GSH) production. High GSH production is important for maintenance of pluripotency because glutamine-derived GSH plays as a scavenger for ROS and prevents OCT4 degradation. Moreover, glucose-mediated cytosolic acetyl-coenzyme A (CoA) contributes to histone acetylation for pluripotency as well as to lipid synthesis for proliferation, while threonine- or methionine-derived SAM also contributes to histone H3K4me3, which is also important for the maintenance of pluripotency in PSCs. In contrast to PSCs, differentiated cardiomyocytes can utilize pyruvate or lactate efficiently for ATP production and GSH synthesis. PPP pentose phosphate pathway; NNMT nicotinamide N-methyltransferase; GSH reduced glutathione; SAM S-adenosylmethionine; ROS reactive oxygen species

mPSCs have been reported to be highly dependent on glycolysis [21, 22] (Fig. 1). Specifically, in the process of reprogramming mouse embryonic fibroblasts into miPSCs, the degree of dependence on glycolysis greatly increases and that on oxidative phosphorylation (OXPHOS) decreases, similar to the high dependence of mouse ESCs (mESCs) upon glycolysis. This finding demonstrates that metabolic reprogramming also occurs during the process of iPSC generation. Interestingly, glycolytic gene expression was found to precede pluripotent marker induction during reprogramming [23]. In fact, inhibition of glucose metabolism decreased the reprogramming efficiency. Given the activated glycolytic features of mESCs, it is reasonable that they would be efficiently reprogrammed under a hypoxic condition [24]. These findings indicated that glucose metabolism plays a key role during reprogramming. In addition, during the reprogramming process, a transient OXPHOS burst occurs by induction of estrogen-related nuclear receptors such as ERRα and ERRγ and their co-factors such as PGC1α and PGC1β [25]. By contrast, depletion of these factors reduces the reprogramming efficiency in mouse embryonic fibroblasts [25]. Moreover, the pentose phosphate pathway (PPP) and synthesis of amino acids are also activated in mESCs because of the need to produce nucleotides and amino acids for proliferation [26••]. Taken together, these studies suggest that mPSCs are dependent on glycolysis not only for ATP production, but also for biomass production, including nucleotides and amino acids. Furthermore, redox status is also important for the maintenance in PSCs. Yanes et al. demonstrated that mESCs were characterized by a high reduced-to-oxidized glutathione ratio and abundant metabolites with highly unsaturated structures. As a result, inhibition of the eicosanoid signaling pathway, a well-known pro-oxidative cascade, could promote pluripotency by maintaining the levels of unsaturated fatty acids [27•].

Pluripotency shows diverse states, and each state has different metabolic characteristics. mESCs are typically cultured in medium containing serum plus leukemia inhibitory factor (LIF), which maintains the cells in a naïve state of pluripotency, although they can switch to activin/nodal-dependent epiblast stem cells (EpiSCs) whose state (primed state) is a little downstream from a naïve state. EpiSCs are highly glycolytic and have low mitochondrial respiratory capacity compared to mESCs due to the increased expression of hypoxia-inducible factor 1α [28] (Fig. 1). In addition, transcription factor STAT3, downstream of the LIF-dependent pathway, has been reported to increase OXPHOS in mESCs [29]. mESCs have also been reported to reach a ground state of pluripotency under specific conditions when cultured in medium containing glycogen synthase kinase 3 (GSK3) and mitogen-activated protein kinase (MAPK)/extracellular signal-related kinase (ERK) inhibitors (2i) plus LIF [8]. As the ground-state mESCs (cultured in 2i plus LIF) maintain a high α-ketoglutarate (αKG) level, which is produced from glucose and glutamine, they could readily proliferate in the absence of glutamine. By contrast, mESCs (cultured in serum plus LIF) could not proliferate without glutamine, because αKG is mainly produced from glutamine [30••]. The ground-state mESCs (cultured in 2i plus LIF) exhibited an elevated αKG-to-succinate ratio, which promoted histone and DNA demethylation, including histone 3 lysine 27 trimethylation (H3K27me3) and ten-eleven translocation (Tet)-dependent DNA demethylation. Moreover, pluripotency was maintained because αKG is a cofactor of Fe (II)- and αKG-dependent dioxygenases, including Jumonji C domain-containing histone demethylases and Tet. Recently, the intracellular αKG level was reported to be affected by phosphoserine aminotransferase 1 (PSAT1), an enzyme involved in de novo serine synthesis that utilizes glutamate as an amino group donor and produces αKG in mPSCs. In the undifferentiated state, OCT4, SOX2, and NANOG bind to the PSAT1 enhancer region to regulate PSAT1 expression [31•]. Moreover, mESCs are also highly dependent on threonine catabolism, which produces glycine and acetyl-CoA via threonine dehydrogenase (TDH) that are required for S-adenosylmethionine (SAM) synthesis [32••, 33•]. As SAM production is important for histone methylation, cell culture in threonine-depleted conditions leads to a decrease in the SAM level, which, in turn, reduces the degree of histone H3 lysine 4 trimethylation (H3K4me3), resulting in difficulty of pluripotency maintenance.

#### Metabolism in hPSCs

hPSCs are also dependent on glycolysis for ATP generation, and show decreased pyruvate oxidation due to the high expression of uncoupling protein 2 (UCP2), a mitochondrial inner membrane protein [34]. hPSCs also highly consume several amino acids, including glutamine and methionine. We demonstrated that glutamine oxidation was indispensable for ATP generation in hPSCs, and glutamine metabolism was more strongly activated under glucose-depleted conditions [35•] (Fig. 1). In addition, glutamine metabolism contributes to reduced GSH synthesis. GSH is not only essential to maintain the redox state but also prevents the degradation of OCT4 [36]. Unlike mPSCs, hPSCs are not dependent on threonine catabolism, because the human *TDH* gene is a nonfunctional pseudogene and threonine cannot contribute to SAM production. Instead, hPSCs depend on methionine catabolism for SAM production [37•]. Moreover, hPSCs utilize glucose for the production of cytosolic acetyl-CoA, which promotes histone acetylation in a pluripotent state [38•]. Recently, hPSCs were reported to be able switch to a naïve state by use of a combination of small molecules such as GSK3 inhibitor (CHIR99021), MAPK/ERK kinase (MEK) inhibitor (PD0325901), c-Jun N-terminal kinase (JNK) inhibitor (SP600125), p38 inhibitor (BIRB796), human LIF, insulin-like growth factor (IGF), and basic fibroblast growth factor (bFGF) [9–11]. Sperber et al. demonstrated that nicotinamide N-methyltransferase (NNMT) was upregulated in naïve hPSCs [39]. In naïve hPSCs, NNMT consumes SAM, which leads to maintenance of low SAM levels and the H3K27me3-repressive state.

### Manipulation of Pluripotent Stem Cell Metabolism

Glucose, glutamine, and methionine metabolism are indispensable for the self-renewal, pluripotency, and survival of PSCs in terms of their contributions to energetics, epigenetics, and redox status. Therefore, it follows that manipulation of glucose, glutamine, and methionine metabolism can be used to regulate the differentiation efficiency and survival of PSCs.

#### Differentiation and Metabolism

Several studies have demonstrated the essential roles of cellular metabolism during the differentiation of PSCs. Upon differentiation, PSCs show reduced reliance on glycolysis and increased mitochondrial numbers and maturation [40], leading to repression of UCP2 expression and a consequent increase in oxidative phosphorylation and reactive oxygen species (ROS) generation [41, 42]. It is well known that ROS enhance the differentiation efficiency of hESCs into cardiomyocytes via activation of p38 MAPK and/or phosphoinositol 3-kinase [43, 44]. A supra-physiological concentration of glucose in the culture medium was shown to result in increased ROS production, leading to enhanced cardiomyocyte differentiation [45]. Intriguingly, supplementation of hydrogen peroxide was shown to improve cardiogenesis in low glucose conditions. These findings were also supported by the fact that mESCs show abundant intracellular polyunsaturated fatty acids, which decrease after differentiation. As the ROS level is increased during differentiation, unsaturated fatty acids are oxidized, leading to an increased eicosanoid level. Therefore, its downstream oxidized metabolites such as palmitic acid, capric acid, and palmitoyl carnitine promote the differentiation of mESCs into neurons or cardiomyocytes [27•]. In addition, supplementation of ascorbic acid could enhance cardiomyocyte differentiation from PSCs [46, 47]. Although ascorbic acid is known for its antioxidant property, other antioxidants such as N-acetylcysteine or vitamin E failed to recapitulate the observed positive effects of ascorbic acid on differentiation. Ascorbic acid was also reported to promote cardiogenesis via induction of the proliferation of cardiac progenitor cells through increased collagen synthesis via the MEK-ERK1/2 pathway [46, 48]. Together with the findings that ROS enhance the PSC differentiation efficiency, these results suggest that ascorbic acid might have a specific effect other than modulation of the redox status [47].

Decreased glycolysis was shown to reduce the levels of cytosolic acetyl-CoA, which is utilized for histone acetylation. The need for the reduction of acetyl-CoA in differentiation was confirmed by supplementation of its precursor acetate, which blocks early histone deacetylation and delayed differentiation [38•]. In addition, although a high αKG level is important for maintenance of pluripotency via histone and DNA demethylation in mPSCs, the intracellular αKG level was shown to decline transiently during differentiation, whereas αKG supplementation delayed differentiation in mPSCs [31•]. By contrast, TeSlaa et al. reported that αKG supplementation promoted the early differentiation of hPSCs, while accumulation of succinate delayed differentiation [49•]. Moreover, another group demonstrated that glutamine deprivation led to differentiation into endothelial cells from hPSCs because glutamine-derived GSH is essential to prevent the degradation of OCT4 [36]. The interpretation of these findings in hPSCs is slightly complicated, because in some cases, glutamine-derived αKG promoted differentiation, whereas in other cases, it was delayed. Hence, further studies are needed to clarify the relationship between differentiation and glutamine metabolism. However, in human hematopoietic stem cells, glucose and glutamine metabolism are known to be essential for erythroid differentiation through de novo nucleotides synthesis. Blocking glucose and glutamine metabolism or nucleotides synthesis inhibited erythroid commitment, even in the presence of erythropoietin, because of the lack of nucleotides [50•]. Furthermore, methionine affects the epigenetic status in hPSCs via SAM production, as described above. Thus, exposure to methionine deprivation could rapidly reduce the intracellular SAM level, leading to the reduction of H3K4me3 and DNA methylation, followed by activation of p53-38 signaling and suppression of NANOG expression [37•]. Indeed, short-term methionine deprivation resulted in the promotion of differentiation into all three germ layers.

#### Elimination of Residual PSCs

Despite great improvements in methods for achieving the differentiation of PSCs to target cells, it is nearly impossible to ensure that all iPSCs can differentiate into target cells. Accordingly, a given population of PSC-derived cells generally contains residual undifferentiated PSCs, which are the main contributors to tumor formation. The risk of tumor formation was reported to be higher with a contamination rate of residual PSCs of more than 0.02% in PSC-derived cells [51, 52]. Therefore, to improve the safety of PSC-based applications, many methods have been developed to eliminate these residual PSCs. Cell sorting is one such method, in which pluripotent markers such as TRA1-60, SSEA-4, or SSEA-5 are used [17]. Although these strategies are simple, the methods are not suitable for large-scale culture because use of FACS is a time-consuming process. Alternatively, a metabolism-based approach is thought to be ideal for large-scale culture in terms of its simplicity, scalability, and low cost. To efficiently eliminate residual PSCs, it is necessary to understand and facilitate the metabolic characteristics of PSCs. As described above, PSCs have an activated glycolysis and PPP. Therefore, by utilizing these characteristics, it is natural that glucose depletion from the culture media will effectively eliminate residual PSCs [26••, 53]. In addition, our group demonstrated that glutamine metabolism contributes not only to nucleotides and GSH synthesis but also to ATP generation [35•]. Consequently, we showed that glucose- and glutamine-depleted conditions efficiently eliminated residual PSCs for short periods [35•]. Moreover, since hPSCs depend on methionine catabolism for SAM production, prolonged methionine deprivation induced the apoptosis of hPSCs via activation of the p53-p38 cell apoptosis signaling pathway [37•]. However, these specific conditions may affect the target cells if PSCs are cultured under the conditions for long periods, because such conditions are based on the deprivation of essential metabolites for PSCs. Therefore, other alternative metabolites should be applied to minimize the negative effects.

#### Metabolic Selection of Differentiated Cardiomyocytes

To identify alternative metabolites for target cells, it is necessary to first understand their metabolism. For example, our group focused on PSC-derived differentiated cardiomyocytes that displayed a fetal phenotype. In the fetal heart, glucose and lactate are the major energy substrates because the concentrations of both glucose and lactate are high in the fetal circulation [54, 55]. Therefore, we hypothesized that lactate would be an alternative energy source under glucose-depleted conditions for PSC-derived cardiomyocytes. In fact, the PSCs could efficiently utilize lactate for ATP generation [26••]. Moreover, lactate was also found to contribute to glutamate and glutamine synthesis under glutamine-depleted conditions [35•]. Interestingly, hPSCs cannot efficiently utilize pyruvate or lactate due to the poor expression of metabolic enzymes such as aconitase 2 and isocitrate dehydrogenase 2/3, in contrast to differentiated cardiomyocytes [35•] (Fig. 1). Furthermore, most of the hPSC-derived non-cardiac cells remaining after differentiation also depend on glucose and/or glutamine and show difficulty in the utilization of lactate [35•]. As a result, we succeeded in purifying only differentiated cardiomyocytes by cultivation under glucose- and glutamine-depleted conditions with lactate supplementation. After purification, contamination of residual PSCs was lower than 0.001%. Therefore, metabolically selected cardiomyocytes derived from hPSCs appear to be safer for transplantation. To date, the strategies developed for the purification of cardiomyocytes have been based on a combination of FACS and the use of antibodies or mitochondrial dye [40, 56]. Therefore, adoption of a metabolic approach will represent a breakthrough in the large-scale production of hundreds of millions of cardiomyocytes for transplantation without requiring an enormous cost.

## Conclusions

Metabolism plays many roles in the reprogramming, pluripotency, self-renewal, differentiation, and survival of PSCs in terms of regulating their energetics, epigenetics, and redox status. Therefore, understanding and manipulating the metabolic regulation mechanisms of PSCs should prove to be a useful strategy for enhancing their beneficial properties and safety for clinical application. Indeed, emerging evidence shows that metabolic regulation by supplementation of certain metabolites or compounds can delay or promote differentiation. Moreover, the use of specific culture conditions can selectively eliminate residual undifferentiated PSCs and purify the differentiated cardiomyocytes. As described above, metabolic approaches have several advantages over conventional methods for PSC reprogramming and differentiation, including their simplicity, scalability, and low cost. Our group has already successfully established large-scale culture methods to obtain a large number of metabolically selected cardiomyocytes with low cost (unpublished). The main challenge in this field at present is to develop a comprehensive understanding of the metabolic profiles in PSCs, which will certainly lead to the development of new technologies to efficiently produce a large number of target cells for clinical application.
